# Generation of high photocurrent in three-dimensional silicon quantum dot superlattice fabricated by combining bio-template and neutral beam etching for quantum dot solar cells

**DOI:** 10.1186/1556-276X-8-228

**Published:** 2013-05-15

**Authors:** Makoto Igarashi, Weiguo Hu, Mohammad Maksudur Rahman, Noritaka Usami, Seiji Samukawa

**Affiliations:** 1 Institute of Fluid Science, Tohoku University, 2-1-1 Katahira, Aoba, Sendai 9808577, Japan; 2 Japan Science and Technology Agency, CREST, 5 Sanbancho, Chiyoda, Tokyo 1020075, Japan; 3 Institute for Materials Research, Tohoku University, 2-1-1 Katahira, Aoba, Sendai, 9808577, Japan; 4 WPI Advanced Institute for Materials Research, Tohoku University, 2-1-1 Katahira, Aoba, Sendai, 9808577, Japan

**Keywords:** Si nanodisk, Aspect ratio, Photocurrent, Miniband

## Abstract

We fabricated a three-dimensional (3D) stacked Si nanodisk (Si-ND) array with a high aspect ratio and uniform size by using our advanced top-down technology consisting of bio-template and neutral beam etching processes. We found from conductive atomic microscope measurements that conductivity became higher as the arrangement was changed from a single Si-ND to two-dimensional (2D) and 3D arrays with the same matrix of SiC, i.e., the coupling of wave functions was changed. Moreover, our theoretical calculations suggested that the formation of minibands enhanced tunneling current, which well supported our experimental results. Further analysis indicated that four or more Si-NDs basically maximized the advantage of minibands in our structure. However, it appeared that differences in miniband widths between 2D and 3D Si-ND arrays did not affect the enhancement of the optical absorption coefficient. Hence, high photocurrent could be observed in our Si-ND array with high photoabsorption and carrier conductivity due to the formation of 3D minibands.

## Background

Quantum dot superlattices (QDSLs) have attracted a great deal of interest from both physical scientists and device researchers. Electron wave functions diffuse and overlap, which merge discrete quantum levels into minibands, with quantum dots approaching and forming a quasi-crystal structure. This band rearrangement has significant applications for many novel optoelectronic/electronic devices [1-15]. For example, quantum dot solar cells, the most exciting photovoltaic device with more than 63% conversion efficiency, have to utilize minibands for carrier transport and additional optical transitions.

Ideal QDSLs present a great challenge to current nanotechnologies. Several technologies (e.g., chemical solution methods and molecular beam epitaxy (MBE)) have convincingly been used to fabricate relatively uniform quantum dots; however, very few technologies can finitely arrange QDs to form a quasi-crystal structure. The well-developed MBE technology can only achieve very limited control on the direction of growth, which induces a mixed state with the wetting layer. The most direct idea is to develop a top-down nanotechnology. However, nanometer-order sizes exceed most light/electron beam limitations, and suitable masks seem impossible to create. The neutral beam (NB) etching and ferritin bio-template we developed have recently brought about a great breakthrough in that we successfully fabricated two-dimensional (2D) array Si nanodisks (Si-NDs) with sub-10 nm, high density (>10^11^ cm^-2^), and quasi-hexagonal crystallization [16-20].

Photovoltaic conversion efficiency was determined by light absorbance and carrier collection efficiency. Our previous work has proven that wave function coupling relaxes the selection rule to induce additional optical transitions [21,22]. We first observed enhanced conductivity in 2D and three-dimensional (3D) array Si-NDs with a SiC matrix in this study. Moreover, we calculated electronic structures and current transport, which theoretically suggested that minibands enhanced conductivity, within envelope function theory and the Anderson Hamiltonian method. These enhanced optical and electrical properties indicated a potential application for the highly efficient quantum dot solar cells.

## Methods

**Figure 1:**
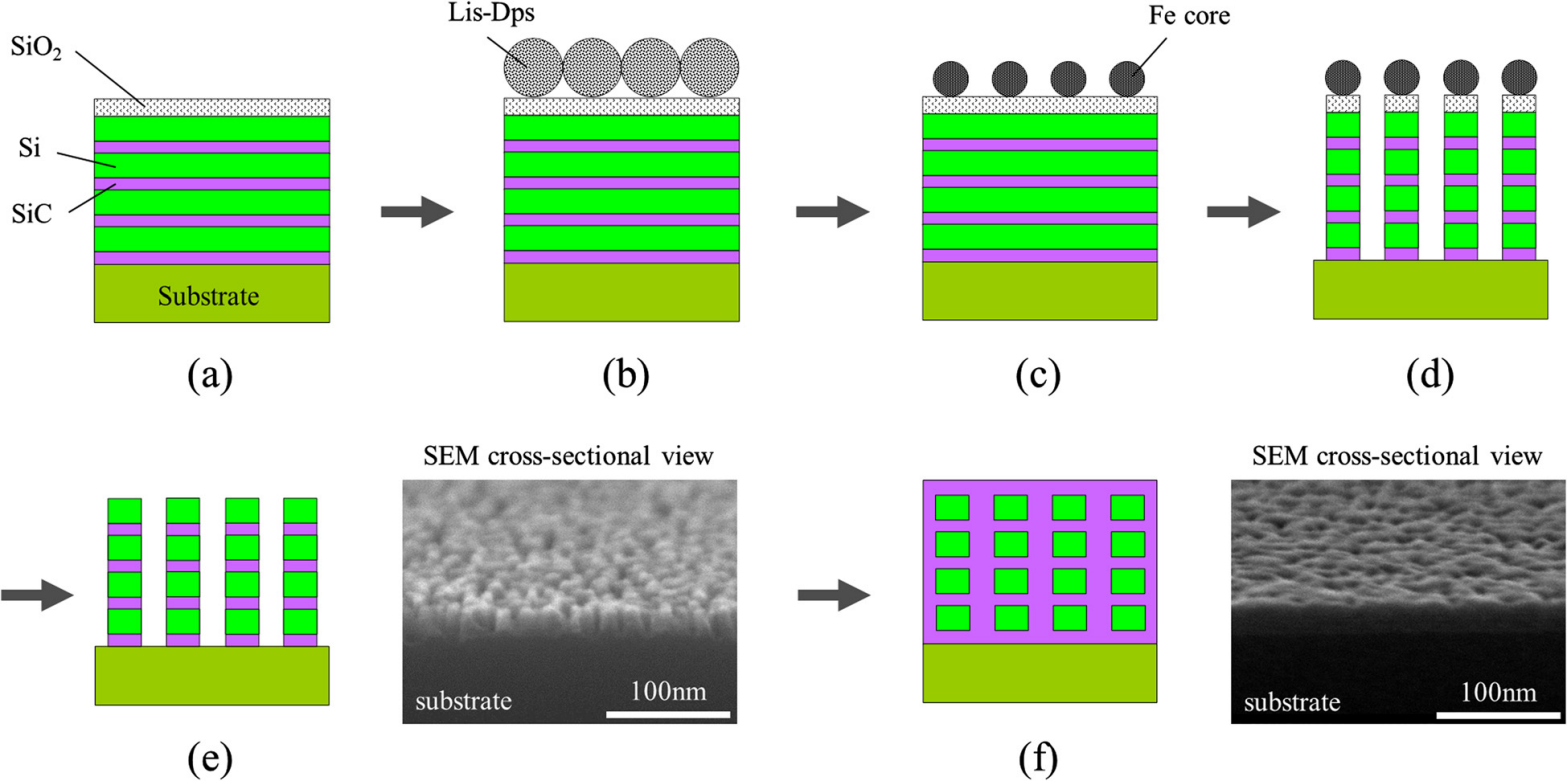
**Schematic of the fabrication flow for 3D array of Si-NDs with SiC interlayer. **(**a**) Deposition of 2-nm-thick SiC, 4-nm-thick poly-Si, and 3-nm-thick SiO_2_ layers. (**b**) Arrangement of 2D array of bio-template molecules on the surface. (**c**) Removal of bio-template protein shell by annealing in oxygen atmosphere. (**d**) NF_3 _treatment to remove surface SiO_2 _and NB etching to remove surface multilayers of poly-Si and SiC. (**e**) Removal of iron cores with HCl and NF_3_ treatment to etch remaining surface SiO_2_. (**f**) SiC deposition on Si-NDs.

**Figure 2:**
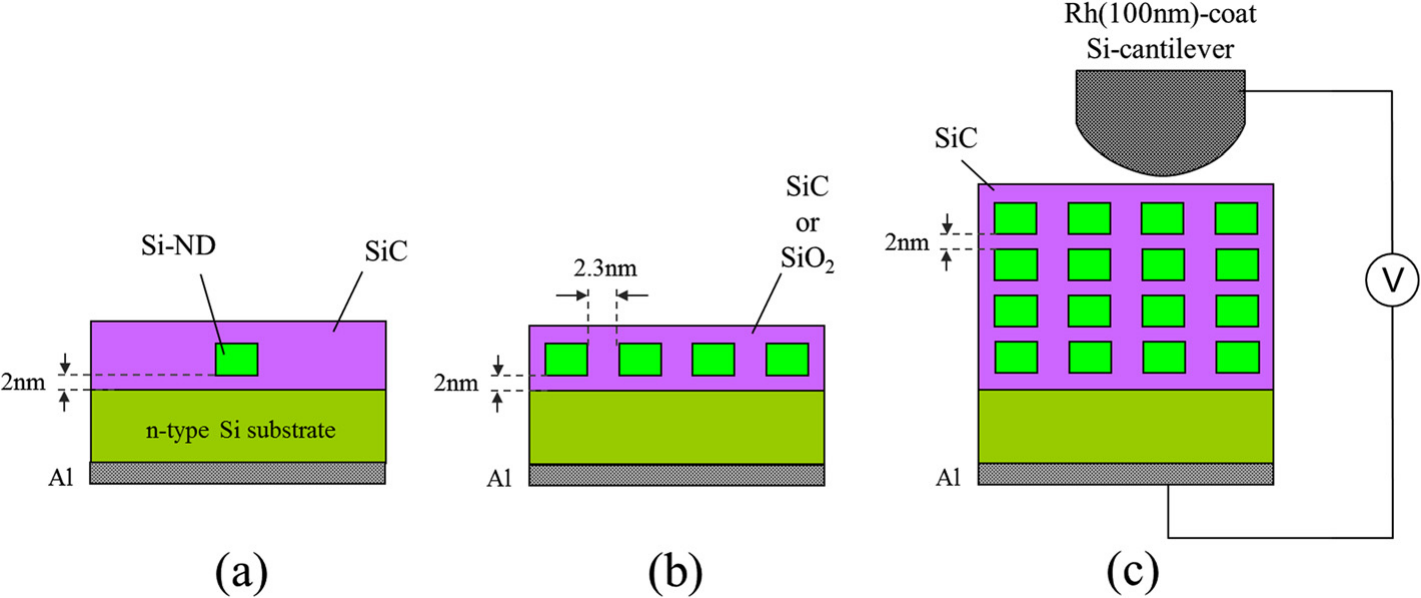
**Schematics of the three types of Si-ND arrangements. **(**a**) Separated Si-NDs as single QD, (**b**) 2D array of Si-NDs as 2D QDSL, and (**c**) 3D array of Si-NDs as 3D QDSL.

The fabrication of the 3D Si-ND array was based on bio-template and NB processes. Figure 1 schematically illustrates the fabrication flow, which started with (Figure 1a) a 2-nm-thick SiC film and 4-nm-thick poly-Si being deposited alternately four times on the n-doped Si substrate using a high-vacuum sputtering system and electron beam evaporation. Then a 3-nm-thick SiO_2_ layer was fabricated as a surface oxide (called NBO-SiO_2_ after this) by the NB oxidation process we developed at a low temperature of 300°C [16]. Figure 1b has a 2D array of bio-template molecules (Listeria-Dps) that was deposited on the surface of the NBO-SiO_2_. Figure 1c shows the bio-template protein shell that was removed by annealing it in an oxygen atmosphere to obtain a 2D array of iron cores as a uniform mask for the etching process. Figure 1d shows the etching process that was carried out with nitrogen trifluoride gas/hydrogen radical treatment (NF_3_ treatment) to remove the surface SiO_2_, which was carried out with NB etching to remove the poly-Si. Here we performed a one-step etching and found a well-aligned vertical etching profile due to high etching selectivity between the iron cores and etched material and the low selectivity of 1.3 between Si and SiC. The etching process has been detailed elsewhere [17-19]. Figure 1e shows that the iron cores were then removed by HCl wet cleaning, and then the remaining surface SiO_2_ was removed by NF_3_ treatment. Figure 1f shows that the SiC was deposited between pillars, which were stacked Si-NDs, by the sputtering system. The diameter, space between NDs, and average ND center-to-ND center distance corresponded to 6.4, 2.3, and 8.7 nm in the structure. The size distribution of the Si-NDs was less than 10% for all samples [19,21]. We prepared three types of Si-ND arrangements, as seen in Figure 2: separated Si-NDs as a single QD, a 2D array of Si-NDs as a 2D QDSL, and a 3D array of Si-NDs as a 3D QDSL. The electrical conductivity and optical absorption in QDSLs were methodically, experimentally, and theoretically investigated with these samples to study the effect of wave function coupling between QDs.

## Results and discussion

**Figure 3:**
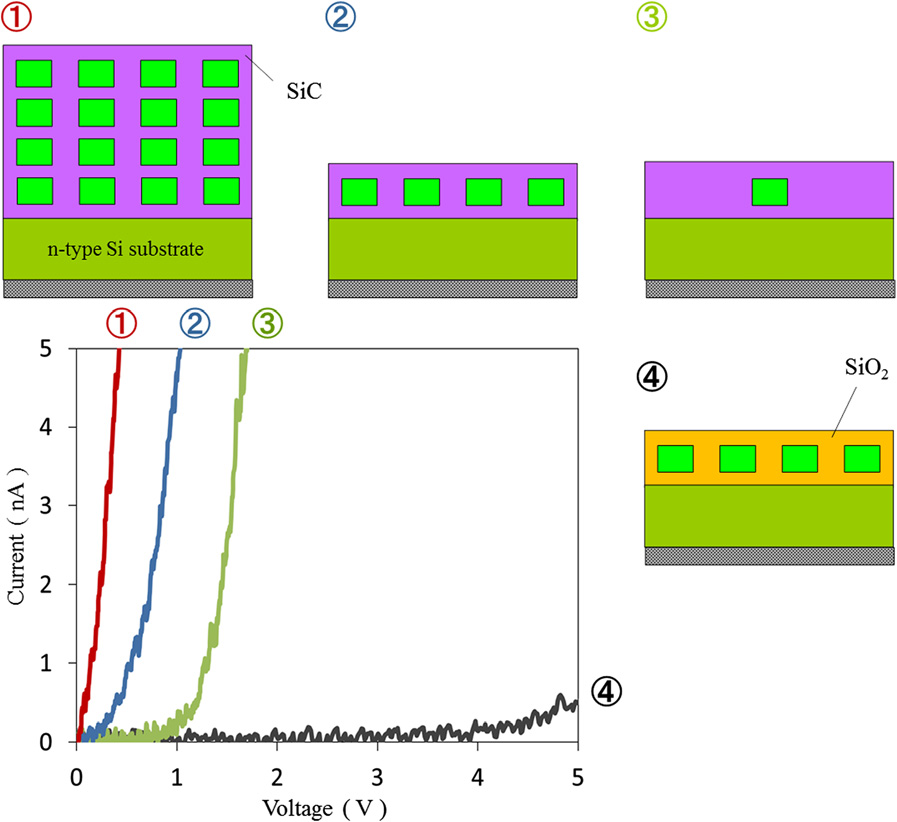
***I*-*V *curves of single Si-ND, 2D, and 3D arrays of Si-NDs measured by c-AFM. **Red, blue, and green lines plot results for the 3D array, 2D array, and single Si-ND with SiC matrix. Black line plots the results for 2D array Si-NDs with SiO_2 _matrix.

Conductive atomic force microscopy (c-AFM) has been used to investigate conductivity, as seen in Figure 3. Changing the matrix from SiO_2_ to SiC greatly increases current (*I*) and decreases threshold voltage (*V*), according to comparisons of the 2D arrays of Si-NDs. Although a primary factor should be macro-conductivity differences between SiC and SiO_2_, one cause is minibands that enhance conductivity, which was revealed in a later theoretical simulation. More significantly, conductivity became higher as the arrangement was changed from a single Si-ND to 2D and 3D arrays with the same matrix of SiC, i.e., the coupling of wave functions was changed. Note that conductivity in the 3D array was higher than that in the 2D array, even though the total thickness of the QDSL expanded. These results indicate that the formation of minibands both in-plane and out-of-plane (vertically) might enhance carrier conductivity in QDSLs.

We considered resonant tunneling to be a theoretical mechanism that could explain our experimental results on the basis of these results. Therefore, we theoretically investigated enhanced conductivity due to the formation of minibands. Our developed top-down nanotechnology achieved great flexibility in designing parts for the quantum structure, such as the independently controllable diameter and thickness, high aspect ratio, and different matrix materials. The finite element method duly described the complex quantum structures. The electronic structure and wave function within envelope function theory are presented as.

(1) $$-\nabla \cdot \left(\frac{\hbar^{2}}{2m^{*}}\nabla \phi \right)+\mathrm{V\phi}=\mathrm{E\phi}$$

**Figure 4:**
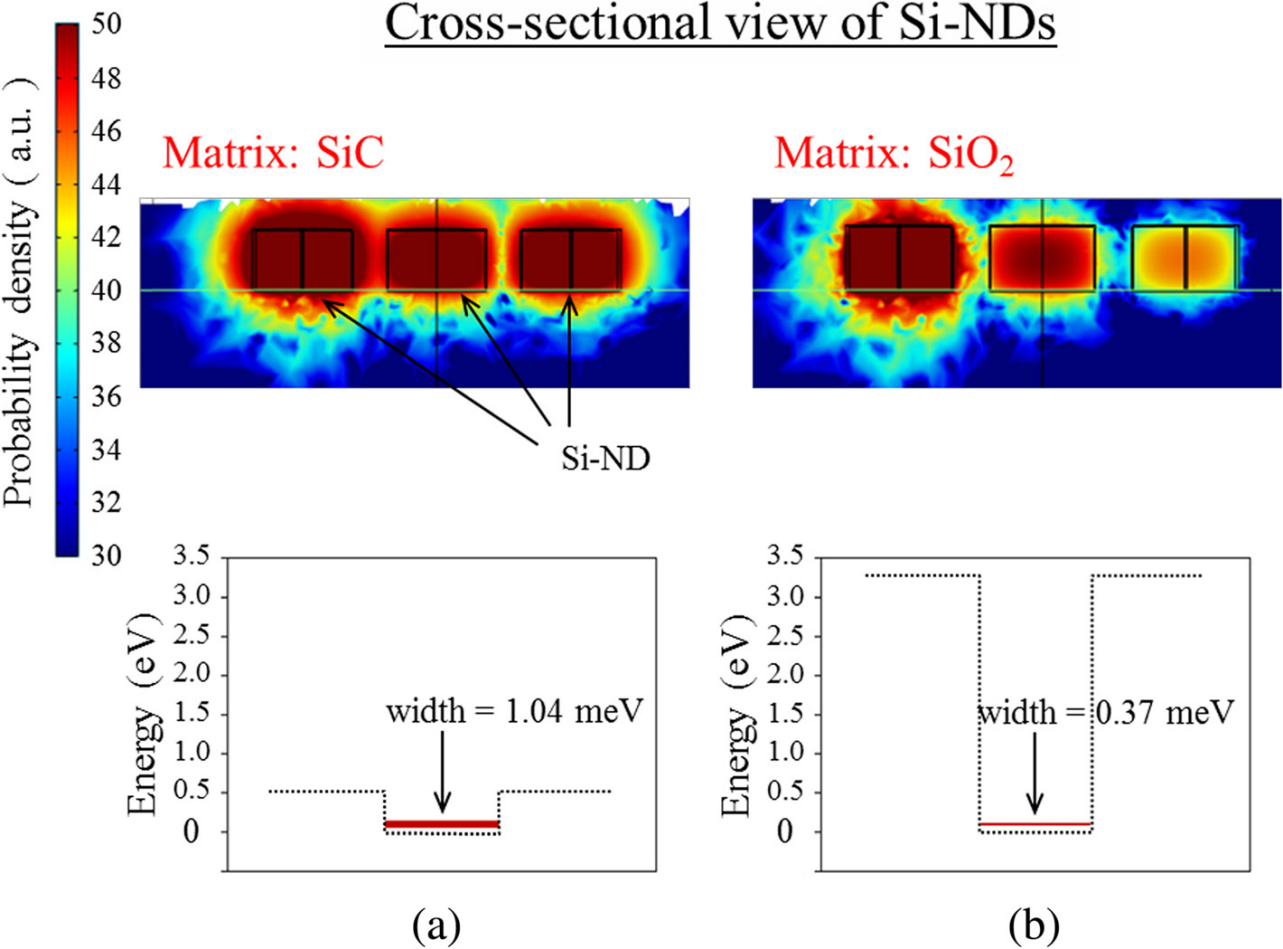
**Calculated results for electron spatial possibilities. **In three lateral coupled NDs and miniband width in 2D array of Si-NDs. Square wave functions and quantum levels of coupled NDs with (**a**) SiC matrix and (**b**) SiO_2 _matrix.

**Figure 5:**
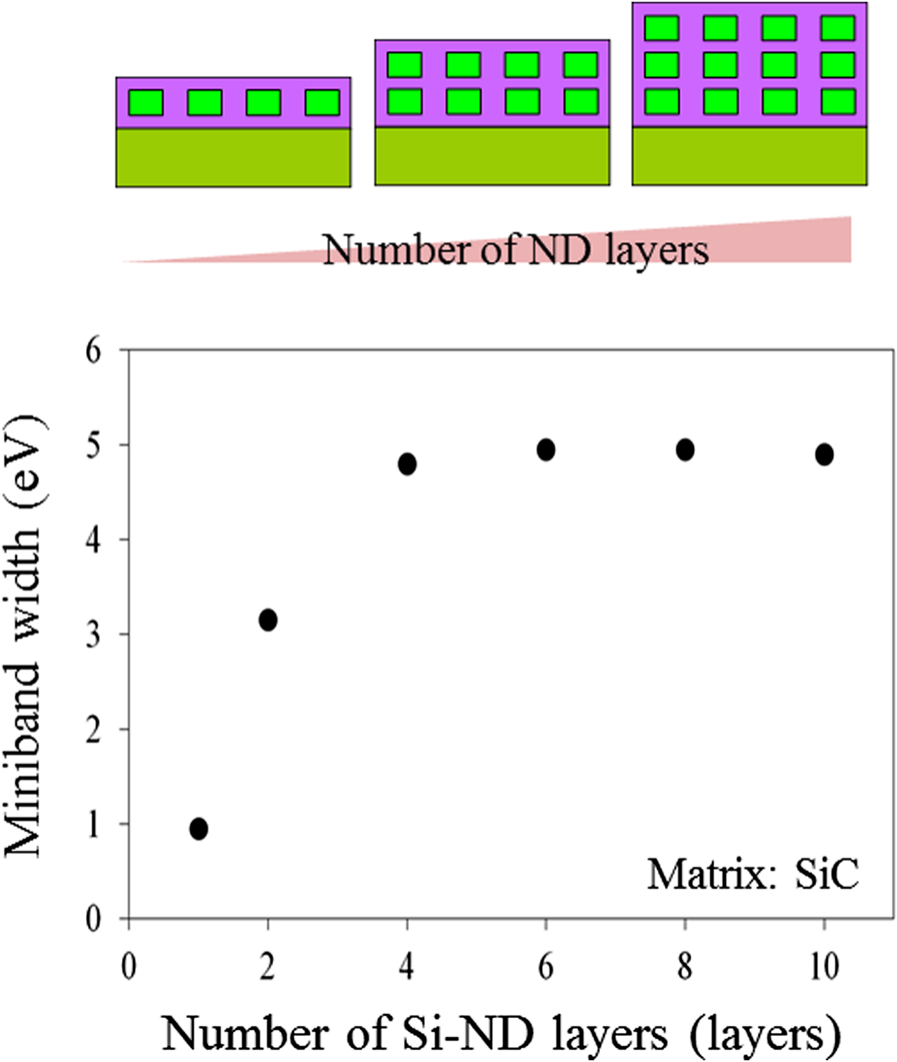
**Calculated results for miniband width in 3D array of Si-NDs. **Thickness, diameter, and space between NDs were assumed to correspond to 4.0, 6.4 and 2.0 nm.

Here we mainly took into consideration the matrix material, realistic geometry structure, and number of stacking layers. The results are presented in Figure 4. A distinct feature is that electron wave functions are more strongly confined in the Si-NDs in the SiO_2_ matrix due to the higher band offset of the Si/SiO_2_ interface. Thus, they resulted in higher quantum levels. In addition, stronger confinement means weaker coupling of the wave function and narrower minibands in the same geometry alignment. By stacking our NDs from one layer to ten layers, the miniband in Figure 5 gradually broadens, and at around four to six layers, the miniband width seems to saturate. The probability of the wave function diffusing into the barrier exponentially reduces with distance, which indicates that wave function coupling exponentially saturates as the number of layers increases. Perhaps four- or six-layer NDs are sufficient to maximize the advantage of minibands.

Chang et al. [23] considered interdot coupling with the Anderson Hamiltonian model to deduce tunneling current density as

(2) $$\begin{array}{l} J=\frac{2\mathrm{eN}}{h}\int_{0}^{\infty }d\epsilon_{z}\int dk_{\mathrm{xy}}\left\{f_{f}\left[\epsilon \left(k\right)-Ef_{f}\right]-f_{d}\left[\epsilon \left(k\right)-Ef_{d}\right]\right\}\cdot \\ \;\frac{\Gamma_{t}\Gamma_{d}}{\Gamma_{t}+\Gamma_{d}}\;\mathrm{lm}\;G_{\sigma }^{\gamma }\left[\epsilon \left(k\right),\;E\left(k_{\mathrm{xy}}\right)\right] \end{array}$$

**Figure 6:**
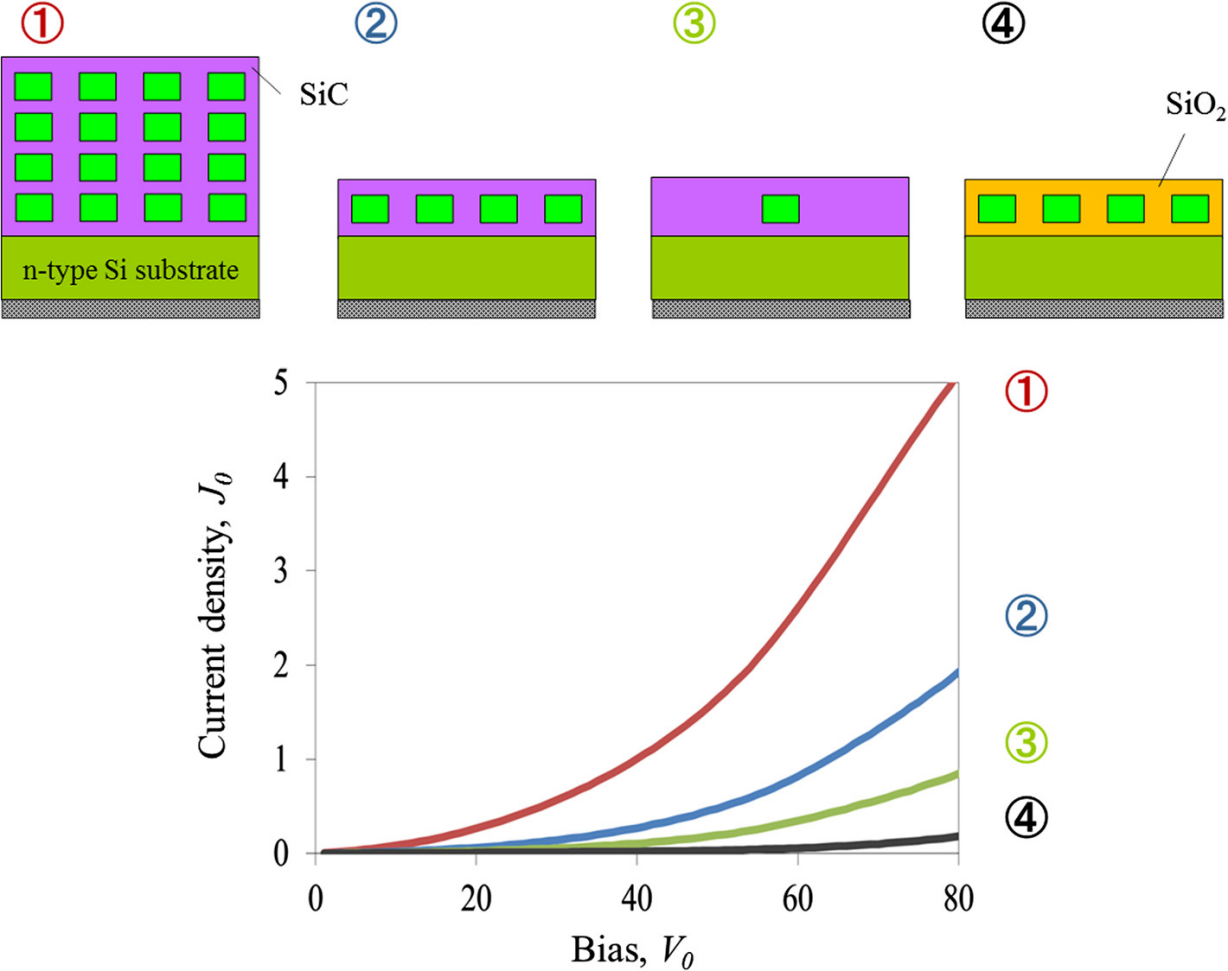
**Simulation results for *I*-*V *properties of our sample structures. **Red, blue, and green lines plot calculated results for 3D array, 2D array, and single Si-ND with SiC matrix. Black line plots results for 2D array Si-NDs with SiO_2 _matrix.

Here *E*(*k*_*xy*_) is related to the energy discrepancy, *t*, due to in-plane ND coupling *E*(*k*_*xy*_) = 2*t*[cos(*k*_*x*_*R*) + cos(*k*_*y*_*R*)]. We simulated the *I*-*V* properties of our structures with this. The results are in Figure 6. The calculated results also revealed that the wider minibands in the SiC matrix resulted in better transport properties than those in the SiO_2_ matrix. A simplified, but not too obscure, explanation is that the formation of minibands broadens the resonance levels to increase joint-state density. Carrier transport in this two-barrier structure mainly depends on resonant tunneling. Moreover, if the Coulomb blockade effect is neglected, the tunneling joint-state density in Equation 2 can be simplified as a parabola function with a resonant peak at ~*E*_0_*– E*(*k*_*xy*_). The formation of minibands broadens the resonant peak to allow more states to approach maximum, which results in enhanced current. Thus, wider minibands mean a higher current density and lower threshold voltage, as can be seen in the Si-NDs in the SiC matrix. In addition, the 2D array of Si-NDs in the SiC matrix has a lower miniband level, *E*_0_, which also shifts the *I*-*V* curves to a lower threshold voltage. This tendency closely matches that in our experimental results, and due to the larger tunneling resistance in the SiO_2_ interlayer (*C*_*t*_), the threshold voltage (*V*) is further increased in realistic *I*-*V* curves. Moreover, conductivity in the 2D and 3D arrays of Si-NDs was enhanced due to the same mechanism that broadened the wave functions and formed wider minibands. As these were also very consistent with the trend in our experimental results, they clarified that the formation of minibands both in-plane and out-of-plane could enhance carrier transport in QDSLs. Enhanced conductivity is very important for electronic/optoelectronic devices, which indicates high charge injection efficiency in lasers and carrier collection efficiency in solar cells.

**Figure 7:**
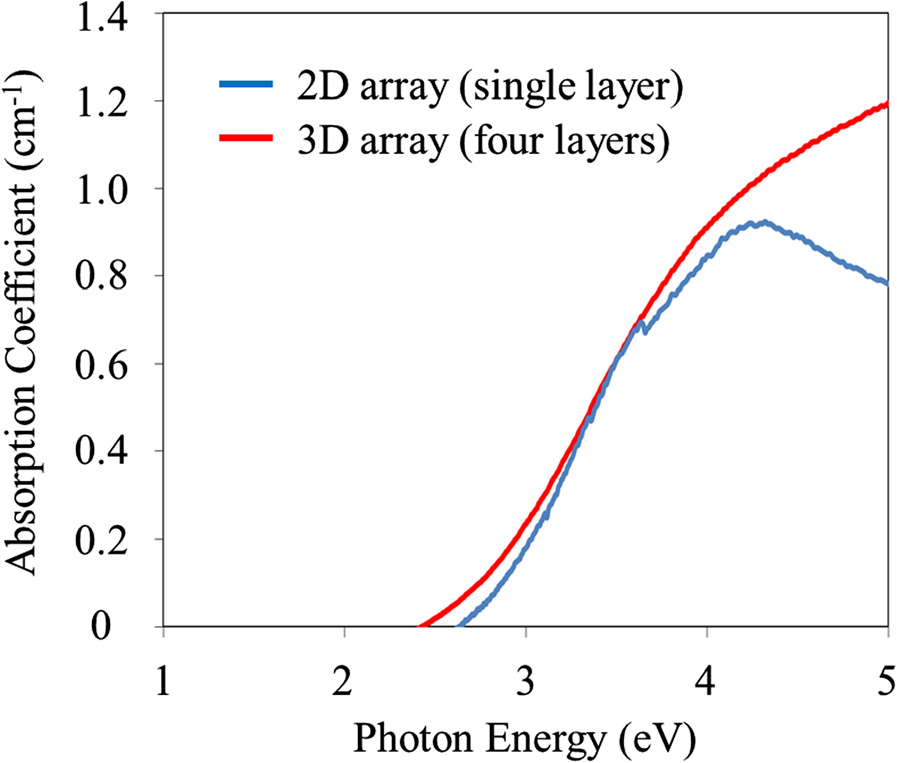
**Absorption coefficients of 2D and 3D arrays of Si-NDs with SiC matrix. **Blue and red lines correspond to 2D and 3D arrays of Si-NDs.

Optical absorption was then investigated by measuring the transmittance of samples using ultraviolet-visible-near-infrared spectroscopy. Our previous work demonstrated that the formation of minibands perpendicular to incident light could enhance photon absorption, i.e., 2D minibands could improve the absorption coefficient in the 2D array of Si-NDs [21,22]. Therefore, we investigated what effect 3D minibands had on optical absorption in this study. Figure 7 shows the absorption coefficients in the 2D and 3D Si-ND array samples prepared on transparent quartz substrates. The absorption coefficient in the 3D array was almost the same as that in the 2D array, and the calculated bandgap energy of both samples was 2.2 eV. Moreover, the change in the miniband width between the samples should be 3.85 meV, as shown in Figure 5 (0.95 meV in single layer and 4.80 meV in four layers). Therefore, it seems that the change of 3.85 meV in the miniband width is not sufficiently large to affect photon absorption.

**Figure 8:**
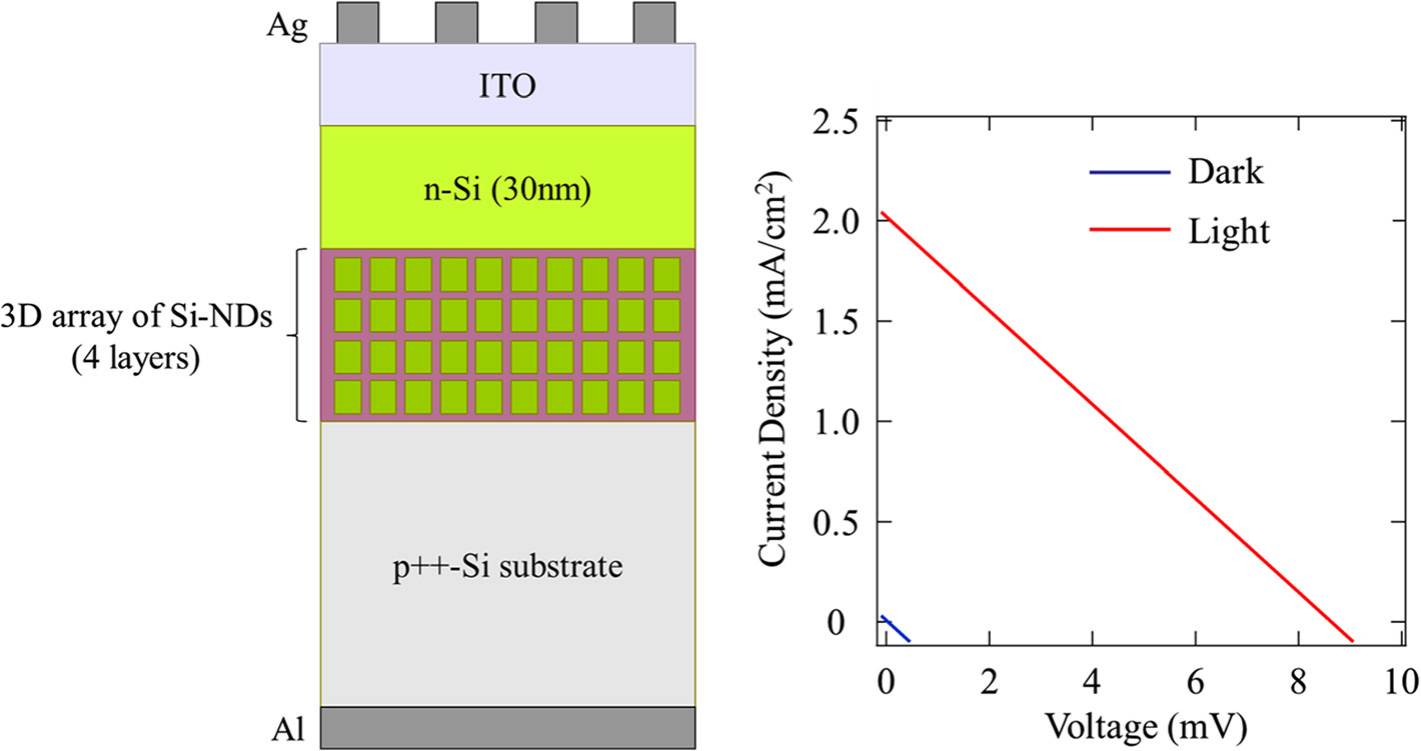
***I*-*V *characteristics of p**^**++**^**-i-n solar cell. **Current-voltage characteristics in dark (blue line) and under sunlight (red line).

Finally, we fabricated a p^++^-i-n Si solar cell with a 3D array of Si-NDs as an absorption layer, as shown in Figure 8, and measured the amount of possible photocurrent generated from the Si-ND layers where the high doping density (>10^20^ cm^-3^) of the p^++^-Si substrate prevented photocurrent from being generated inside the substrate itself. Here we found that the generated short-circuit current density from the p^++^-i-n solar cell was 2 mA/cm^2^, where the largest possible photocurrent generated in the Si-ND layers and n-Si emitter was estimated to be 3.5 mA/cm^2^ for the former and 1.0 mA/cm^2^ for the latter [22]. Since 1 mA/cm^2^ is the highest possible value for photocurrent from the n-Si emitter according to this estimate, the actual value should be lower than the calculated value. Therefore, we found that out of the total photocurrent of 2 mA/cm^2^, much more of it (>1 mA/cm^2^) was contributed to by Si-ND. This confirms that most of the observed photocurrent originated from the carrier generated at the Si-ND itself because of high photoabsorption and carrier conductivity due to the formation of 3D minibands in our Si-ND array.

## Conclusions

We developed an advanced top-down technology to fabricate a stacked Si-ND array that had a high aspect ratio and was of uniform size. We found from c-AFM measurements that conductivity increased as the arrangement was changed from a single Si-ND to 2D and 3D arrays with the same matrix of SiC. This enhancement was most likely due to the formation of minibands, as suggested by our theoretical calculations. Moreover, the change in out-of-plane minibands did not affect the absorption coefficient. This enhanced transport should work in the collection efficiency of high carriers in solar cells.

## Abbreviations

c-AFM: Conductive atomic force microscopy; I-V: Current-voltage; MBE: Molecular beam epitaxy; ND: Nanodisk; QDSL: Quantum dot superlattices.

## Competing interests

The authors declare that they have no competing interests.

## Authors' contributions

MI and SS conceived and designed the experiment, fabricated the silicon nanodisk samples, performed electrical and optical measurements, analyzed these data, and wrote the paper. MMR and NU fabricated the solar cell structures and analyzed the *I*-*V* data. WH performed the theoretical calculations. All authors discussed the results, commented on the manuscript, and read and approved the final version.
